# Expression Patterns of Circulating MicroRNAs in the Risk Stratification of Bicuspid Aortopathy

**DOI:** 10.3390/jcm9010276

**Published:** 2020-01-19

**Authors:** Evaldas Girdauskas, Niklas Neumann, Johannes Petersen, Tatiana Sequeira-Gross, Shiho Naito, Maria von Stumm, Yskert von Kodolitsch, Hermann Reichenspurner, Tanja Zeller

**Affiliations:** 1 Department of Cardiovascular Surgery, University Heart and Vascular Center Hamburg, 20246 Hamburg, Germany; n.neumann@uke.de (N.N.); joh.petersen@uke.de (J.P.); t.sequeira-gross@uke.de (T.S.-G.); shiho.naito@gmail.com (S.N.); m.stumm@uke.de (M.v.S.); reichenspurner@uke.de (H.R.); 2 German Center of Cardiovascular Research (DZHK), Partner site Hamburg/Lübeck/Kiel, 20246 Hamburg, Germany; kodolitsch@uke.de (Y.v.K.); t.zeller@uke.de (T.Z.); 3 Department of Cardiology, University Heart and Vascular Center Hamburg, 20246 Hamburg, Germany

**Keywords:** biomarkers, circulating microRNAs, aortopathy, bicuspid aortic valve

## Abstract

Objective: Aortic size-based criteria are of limited value in the prediction of aortic events, while most aortic events occur in patients with proximal aortic diameters < 50 mm. Serological biomarkers and especially circulating microRNAs (miRNAs) have been proposed as an elegant tool to improve risk stratification in patients with different aortopathies. Therefore, we aimed to evaluate the levels of circulating miRNAs in a surgical cohort of patients presenting with bicuspid aortic valve disease and distinct valvulo-aortic phenotypes. Methods: We prospectively examined a consecutive cohort of 145 patients referred for aortic valve surgery: (1) Sixty three patients (mean age 47 ± 11 years, 92% male) with bicuspid aortic valve regurgitation and root dilatation (BAV-AR), (2) thirty two patients (mean age 59 ± 11 years, 73% male) with bicuspid aortic valve stenosis (BAV-AS), and (3) fifty patients (mean age 56 ± 14 years, 55% male) with tricuspid aortic valve stenosis and normal aortic root diameters (TAV-AS) who underwent aortic valve+/-proximal aortic surgery at a single institution. MicroRNAs analysis included 11 miRNAs, all published previously in association with aortopathies. Endpoints of our study were (1) correlation between circulating miRNAs and aortic diameter and (2) comparison of circulating miRNAs in distinct valvulo-aortic phenotypes. Results: We found a significant inverse linear correlation between circulating miRNAs levels and proximal aortic diameter in the whole study cohort. The strongest correlation was found for miR-17 (*r* = −0.42, *p* < 0.001), miR-20a (*r* = −0.37, *p* < 0.001), and miR-106a (*r* = −0.32, *p* < 0.001). All miRNAs were significantly downregulated in BAV vs. TAV with normal aortic root dimensions Conclusions: Our data demonstrate a significant inverse correlation between circulating miRNAs levels and the maximal aortic diameter in BAV aortopathy. When comparing miRNAs expression patterns in BAV vs. TAV patients with normal aortic root dimensions, BAV patients showed significant downregulation of analyzed miRNAs as compared to their TAV counterparts. Further multicenter studies in larger cohorts are needed to further validate these results.

## 1. Introduction

Acute aortic syndrome remains the most catastrophic and life-threatening event in patients presenting with hereditary and congenital aortopathies. Measurements of maximal aortic diameter by standard imaging tools such as computed tomography are of limited value in predicting the risk of aortic events (i.e., aortic dissection/rupture) [1]. Only a small patients’ proportion would have met the indication for diameter-based elective aortic replacement prior to the dissection event, according to the most recent guidelines [1,2]. Therefore, circulating biomarkers have been proposed as an elegant tool to improve risk stratification in patients with congenital aortopathies [3]. Circulating microRNAs have been recently suggested as potential biomarkers to identify progressive aneurysmal disease [4]. Since the bicuspid aortic valve (BAV) phenotype might be associated with a congenital aortopathy and an increased risk of aortic events, the identification of microRNAs specifically associated with BAV aortopathy may improve risk stratification in such patients [5]. Herein, we aimed to analyze the expression patterns of circulating microRNAs in patients with bicuspid aortic valve disease and distinct valvulo-aortic phenotypes.

## 2. Methods

### 2.1. Study Population

A total of consecutive 145 patients who were referred for aortic valve surgery with or without concomitant proximal aortic replacement were included in our study. The patients were subdivided into three subgroups, according to the aortic valve morphology and valvulo-aortic phenotypes.

#### 2.1.1. Bicuspid Aortic Valve Root Phenotype Cohort (BAV-AR)

This retrospectively identified study cohort included patients with isolated/predominant bicuspid aortic valve regurgitation and simultaneous aortic root dilatation (i.e., sinus of Valsalva ≥ 40 mm) from our institutional BAV database. Patients with Marfan’s syndrome, concomitant aortic stenosis (mean pressure gradient ≥ 20 mm Hg), or absence of aortopathy (proximal aortic diameter < 40 mm) were excluded from this cohort. After excluding all patients with known connective tissue disorders and those with mixed bicuspid aortic valve disease, we retrospectively identified 63 patients in the BAV-AR cohort.

#### 2.1.2. Bicuspid Aortic Valve Stenosis Cohort (BAV-AS)

Thirty-two consecutive BAV patients from our prospective institutional BAV surgery database underwent an isolated aortic valve replacement (AVR) due to severe bicuspid aortic valve stenosis (i.e., mean transvalvular gradient ≥ 40 mm Hg, orifice area < 1.0 cm²). All patients in the BAV-AS cohort had normal or only mildly dilated ascending aorta (i.e., < 40 mm).

#### 2.1.3. Tricuspid Aortic Valve Stenosis Cohort (TAV-AS)

Fifty consecutive tricuspid aortic valve (TAV) patients from our prospective institutional BAV surgery database underwent an isolated AVR surgery due to severe tricuspid aortic valve stenosis (i.e., mean transvalvular gradient ≥ 40 mm Hg, orifice area < 1.0 cm²) and normal or only mildly dilated ascending aorta (i.e., <40 mm). This patient cohort served as a control group.

### 2.2. Definitions and Measurements

Bicuspid aortic valve was suspected prior to surgery when two-dimensional transthoracic echocardiographic short-axis depicted the existence of only two normal commissures resulting in two aortic valve cusps. Further evaluation was performed using intraoperative transesophageal echocardiography and the final verification was performed by intraoperative description by the surgeon. Further categorization of BAV was determined using the Sievers’ classification [6]. Aortic valve lesions (i.e., stenosis and regurgitation) were quantified using the published echocardiography guidelines [7].

Proximal aortic diameters were assessed by means of transthoracic echocardiography at the level of sinus of Valsalva in the parasternal long axis view using the leading-edge measurements [7]. All patients with a suspected aortic aneurysm or echocardiographic evidence of aortic root diameters ≥ 40 mm underwent preoperative computed tomography or magnetic resonance imaging (MRI). All aortic diameters were measured perpendicular to the central aortic axis in accordance with the previously published guidelines [8].

### 2.3. MicroRNA Analysis

Venous blood samples were obtained prior to surgery, serum was extracted by centrifugation before freezing, and circulating RNA was isolated from frozen serum samples using the PAXgene blood miRNA kit (Qiagen, Hilden, Germany) on a QIAcube system (Qiagen) according to the manufacturer’s recommendations. The RNA concentration was measured on the NanoDrop N1000 System (peqlab), and 10 ng of RNA was used for microRNA analysis. The RNA quality was measured using an Agilent 2100 bioanylizer (Agilent Technology, Inc., Santa Clara, CA, USA). For further analysis, only samples with RNA integrity number above seven were included. We selected our microRNAs from the published literature based on their reported association with BAV- or TAV-associated aortopathy, aortic aneurysm formation, as well as occurrence of aortic-related events [4,9]: MiR-1, miR-17, miR-18a, miR-19a, miR-20a, miR-21, miR-29b, miR-106a, miR-133a, miR-143, and miR-145. cDNA synthesis and microRNA analysis were performed using the TaqMan Advanced miRNA cDNA synthesis kit (Thermo Fischer Scientific) on a 7900 HT real-time system. In the case for CT values ≥ 40, microRNA was considered as undetermined [10].

### 2.4. Statistical Analysis

Continuous variables are expressed as mean ± standard deviation and categorical variables are presented as percentages. All statistical analyses were performed using the IBM SPSS 22.0 software (IBM Corp, New York City, NY, USA). All *p*-values < 0.05 were considered statistically significant. Bonferroni correction was used during multiple comparison testing. One-way ANOVA was used to compare microRNA expression patterns (i.e., comparison of delta CT values of all analyzed microRNAs) between BAV-AR, BAV-AS, and TAV subgroups. Correlation analyses between microRNA values and maximal aortic diameter in the whole study cohort and subgroups were performed using the Pearson correlation coefficient.

## 3. Results

Demographics and baseline variables are summarized in Table 1. The BAV-AR cohort consisted of young and predominantly male patients with aortic root dilatation and BAV regurgitation. BAV-AR patients were significantly younger as compared to BAV-AS patients (47 ± 11.3 vs. 59 ± 9.7 years; *p* < 0.001) and TAV-AS patients (47 ± 11.3 vs. 56 ± 14 years; *p* = 0.001). Arterial hypertension was present in one third of BAV-AR patients and was significantly more common in the BAV-AS (53%), as well as the TAV-AS subgroup (66%). Other comorbidities were comparable among the three study subgroups. During AVR surgery, the largest AVR prosthesis size was used in the BAV-AR cohort (Table 1).

### Biomarker Analysis

Our biomarker analysis included 11 specific microRNAs, all previously reported in association with aortopathies. Four microRNAs were undetectable (miR-1, miR-29b, miR-133a, and miR-143). The remaining seven microRNAs were used for further analyses.

CT values of all analyzed miRNAs were systematically compared among three different valvulo-aortic phenotypes (i.e., BAV-AR vs. BAV-AS vs. TAV-AS), as displayed in Table 2. One-way ANOVA analysis revealed significantly different expression patterns of analyzed circulating microRNAs among the three study subgroups. Although distribution patterns of circulating microRNA CT values were quite heterogeneous between the three subgroups, the microRNAs downregulation was the most obvious in the BAV-AS subgroup (i.e., miR-17, miR-18a, miR-19a, miR-20a, and miR-106a). CT values for miR-145 and miR-21 showed significantly lower levels in the TAV-AS subgroup, whereas the highest CT values were present in the BAV-AR subgroup.

**Figure 1:**
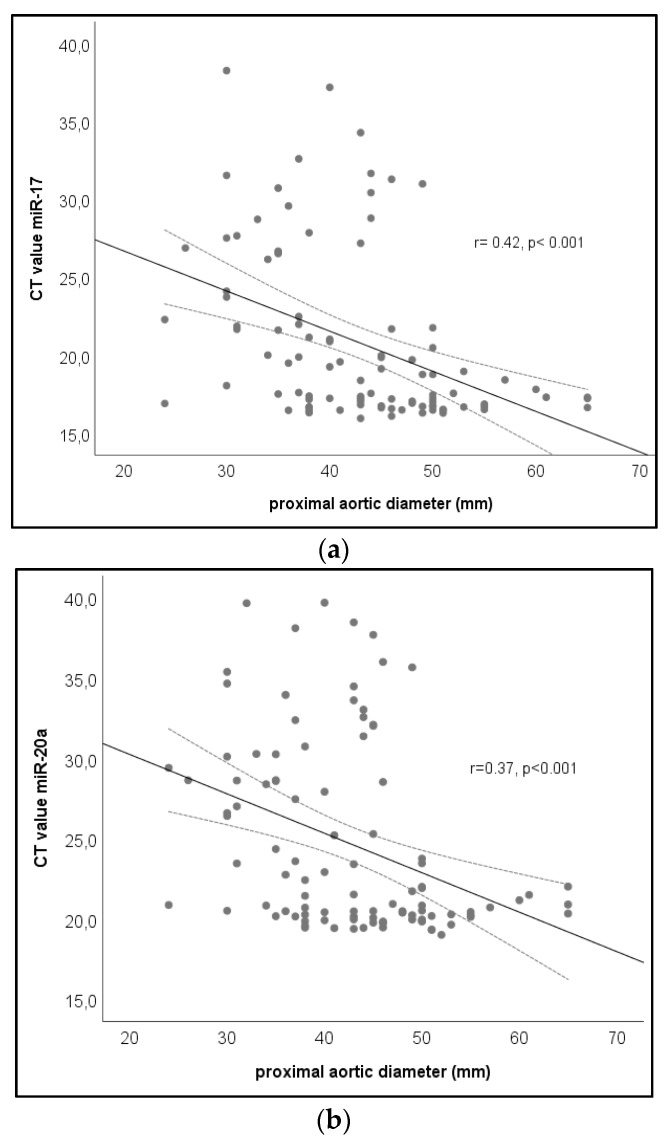
Correlation between aortic diameter and microRNA-17 and microRNA-20a (*n* = 145). (**a**) Scatterplot demonstrating the correlation between maximal aortic diameter and microRNA CT values of miR-17 in the whole study cohort. (**b**) Scatterplot demonstrating the correlation between maximal aortic diameter and microRNA CT values of miR-20a in the whole study cohort.

Correlation analysis between the maximum aortic diameter and circulating microRNA values in the whole study population (*n* = 145) is displayed in Table 3. We found that circulating miR-17 and miR-20a demonstrated a significant inverse linear correlation with the maximum aortic diameter, whereas the remaining microRNAs (i.e., miR-18a, miR-19a, miR-21, mir106a, and miR-145) were not significant in this regard. Inverse linear correlation between the maximum aortic diameter and the circulating CT values of miR-17 (*r* = −0.285; *p* = 0.005) and of miR-20a (*r* = −0.215; *p* = 0.035) is displayed in Figure 1.

**Figure 2:**
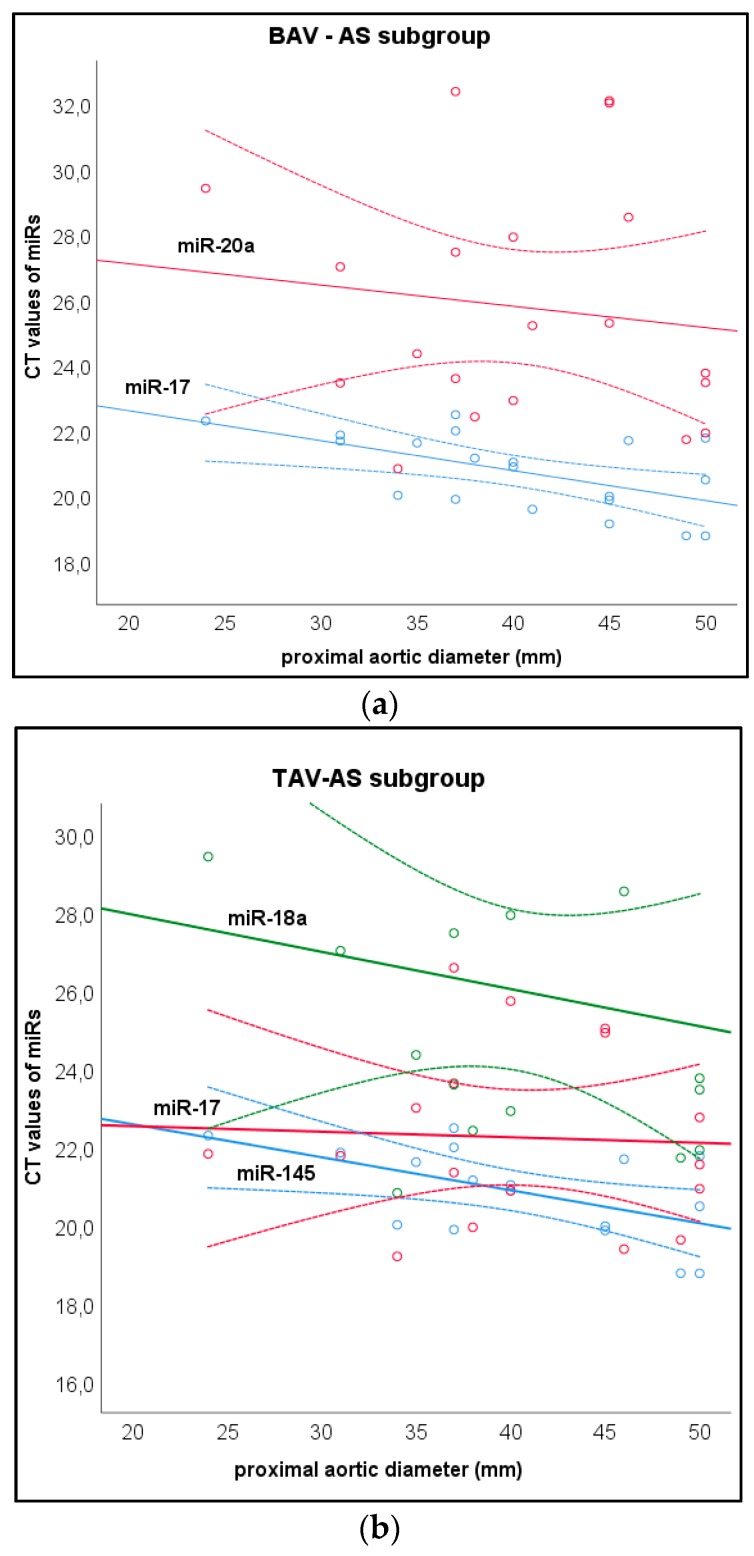
Correlation between microRNAs and aortic diameter in the study subgroups. (**a**) Scatterplot demonstrating the correlation between maximal aortic diameter and microRNA CT values in the Bicuspid Aortic Valve Stenosis (BAV-AS) subgroup (**b**) Scatterplot demonstrating the correlation between maximal aortic diameter and microRNA CT values in the Tricuspid Aortic Valve Stenosis (TAV-AS) subgroup.

To further differentiate the circulating microRNA expression patterns, we analyzed the correlation between the maximum aortic diameter and CT values of circulating microRNAs separately in all three study subgroups (i.e., BAV-AR, BAV-AS, and TAV-AS). Subgroup analysis revealed additionally a significant inverse correlation between maximum aortic diameter and the CT values of miR-18a (*r* = −0.441; *p* = 0.01) miR-145 (*r* = −0.386; *P* = 0.02), and miR-17 (*r* = −0.221; *p* = 0.049) in the TAV-AS subgroup (Figure 2a,b). In the BAV-AS subgroup there was a significant inverse correlation between maximum aortic diameter and the CT values of miR-17 (*r* = −0.479; *p* = 0.01) and miR-20a (*r* = −0.378; *p* = 0.045) (Figure 2a).

Furthermore, considering the fact that all three study subgroups showed significant differences in terms of age, gender, and prevalence of aortopathy (Table 1), we subsequently performed a multivariable linear regression analysis for CT value of each analyzed microRNA in a model that accounted for aortic valve and aortic phenotype, as well as for clinical variables such as age, gender, and proximal aortic dimension. A total of five miRNAs (miR-17, miR-20a, miR-21, miR-106a, and miR-145) showed a significant association with an aortic valve phenotype (i.e., BAV vs. TAV), the presence of aortopathy (i.e., proximal aorta diameter ≥ 40 mm), and the patients’ age. An exemplary multiple linear regression model for CT values of circulating miR-17 is displayed in Table 4.

## 4. Discussion

Conventional imaging modalities have a limited value in the prediction of aortic events in patients with congenital and hereditary aortopathies [1]. Given the fact that the clinical course of aortopathy is frequently occult until dissection or rupture event occurs, remarkable efforts were undertaken to evaluate the impact of circulating biomarkers in the prediction of aortic events.

Previous studies evaluated clinical risk factors [11], annual growth rates of aortic aneurysm [12], or the role of routine biomarkers (e.g., D-dimer) [13] to elucidate the complex pathophysiology of the aortopathy progression. Given the limitations of such conventional tools, circulating microRNAs have been increasingly reported in the association with distinct aortopathies [14].

MicroRNAs are small (i.e., 19–30 nucleotides) noncoding molecules that regulate gene expression at the translational level [15] and exhibit their main action through the translational inhibition of specific messenger RNAs and thereby control diverse cellular functions (i.e., cell proliferation, differentiation, and apoptosis) [16]. Derangements of circulating microRNA have been previously reported in association with various morbid conditions, in particularly malignancies and cardiovascular disorders [16,17].

Due to the fact that progressive aortopathy has been consistently linked to an increased risk of aortic events, we specifically assessed the expression patterns of circulating microRNAs in the surgical cohort of different valvulo-aortic phenotypes (i.e., BAV-AR, BAV-AS, TAV-AS) [18]. Young and predominantly male patients presenting with BAV regurgitation and concomitant aortic root dilatation demonstrate an increased prevalence of genetic abnormalities (i.e., TGFBR2, FBN1, NOTCH1, and SMAD2) [19,20]. As reported previously, such patients potentially have the most malignant aortopathy and, therefore, may present with the most extensive derangements in the microRNA expression [21]. To test this hypothesis, we examined the levels of circulating microRNA in the BAV root phenotype patients (i.e., BAV-AR subgroup) and compared them with other valvulo-aortic phenotypes of aortic valve lesions (i.e., BAV-AS and TAV-AS subgroups).

In all our study, analyzed microRNAs have been previously published in association with aortic aneurysms or specific aortopathies. MiR-1, although undetectable in our analysis, has been previously reported to influence the development of vascular diseases by targeting insulin-like growth factor and thereby regulating the proliferation of vascular smooth muscle cells (VSMC) [22]. Similarly, microRNA-21 has been described to modulate the phosphatase and tensin homolog (PTEN) protein and impact VSMCs proliferation and apoptosis, thereby influencing the development of aortic aneurysms [23]. Furthermore, microRNA-21 seems to play a role in the pathophysiology of atherosclerosis [24]. Likewise, microRNA-145 has been demonstrated to influence the formation of neointimal lesions in the damaged arteries in the rat model [25]. MircroRNA-17 and related gene clusters (i.e., miR-17, miR-18a, miR-19a) have been shown to influence the progression of aortic dilation in BAV-associated aortopathy by regulating tissue inhibitor of matrix metalloproteinase (TIMP-1 and -2) expression [9]. Furthermore, TIMP-2 activity is also directly influenced by microRNA-20a and microRNA-106a expression [26].

Since the scientific evidence supporting the impact of circulating microRNAs in the aortopathy progression is still at a premature stage, the practical impact of herein reported miRNA derangements is limited. We only aim to report significant associations instead of suggesting any causal relationship. Nevertheless, we found relevant differences in the microRNA expression patterns which might be valuable, considering even the limitations of our preliminary study. Inverse linear correlation between proximal aortic diameters and the CT values of circulating microRNAs have been described previously and linked to specific cellular pathways [9,21]. In accordance to these studies, we were able to confirm an inverse linear correlation between maximum aortic diameters and the values of circulating microRNA-17 and microRNA-20a in the pooled study population. Furthermore, post-hoc subgroup comparisons revealed additional microRNA aberrations when comparing different valvulo-aortic phenotypes. These findings are similar to the previous studies which found differential expression patterns of microRNAs (i.e., miR-122, miR-130a, and miR-486) in specific valvulo-aortic phenotypes [27]. Consequently, our findings suggest that the correlation patterns between aortic diameters and circulating microRNAs may differ among the subgroups of underlying valvulo-aortic phenotypes. While an inverse correlation in BAV-AS patients was relevant only for microRNA-18a and microRNA-20a, a significant association was demonstrated in patients with severe tricuspid aortic valve stenosis (TAV-AS) for microRNA-17, microRNA-18a, and microRNA-145.

Recently, Sabatino et al. published a comprehensive bioinformatic analysis examining previously published datasets of microRNA profiles in BAV patients [28]. However, the focus of this bioinformatic analysis was primarily on the clinical evolution of aortic valve function in BAV patients, comparing BAV and TAV patients, with and without aortic valve stenosis. This fact had a significant impact on the selection of included microRNAs. Sabatino et al. selected primarily those microRNAs that are modulated in stenotic BAV/TAV patients and are involved in the calcium, phosphates metabolism, blood coagulation, and platelet activation [28]. On the contrary, we selected microRNAs that were specifically published in association with BAV-associated aortopathy and aortic complications. Furthermore, 12/18 (66%) studies included in their bioinformatic review dealt with the tissue microRNAs expression, while our study primarily focuses on the expression patterns of circulating (blood) microRNAs. These differences in the study design may explain the observed differences in the microRNAs selection.

Even though our findings are promising and confirmed the previously published results, they should be interpreted as very preliminary. Considering the limited sample size and tentative approach, further investigations of larger multicenter cohorts are necessary to validate these promising findings. Recently, novel circulating miRNAs (miR-122, miR-130a, miR-486, and miR-718) were reported in association with BAV and aortopathy which were not included in our analysis [27]. Moreover, there is still no evidence that circulating miRNAs, which are associated with progressive aortopathy, arise specifically from the aortic tissue. Therefore, a simultaneous measurement of circulating miRNAs and tissue miRNAs in the proximal aorta is required to answer this question. Our aim is to address this issue in the upcoming prospective study.

## Figures and Tables

**Table 1:** Demographics and intraoperative variables in the three study cohorts.

Variable	BAV-AR (*n* = 63)	BAV-AS (*n* = 32)	TAV-AS (*n* = 50)	*p*-Value
Mean age (years)	47 ± 11.3	59 ± 9.7	56 ± 14	<0.001
Male gender	58 (92)	22 (70)	26 (55)	<0.001
BSA (m²)	2.01 ± 0.2	1,98 ± 0.2	2.10 ± 0.6	0.496
NYHA class III/IV	10 (43)	10 (31)	26 (52)	0.181
Aortic root diameter (mm)	46 ± 8	40 ± 7	37 ± 7	0.000
Arterial hypertension	6 (30)	17 (53)	33 (66)	0.023
Diabetes	1 (5)	4 (12.5)	7 (14)	0.566
History of smoking	11 (55)	15 (60)	19 (44)	0.42
Mean AVR * prosthesis size (mm)	27.8 ± 1.3	24.8 ± 1.7	24.5 ± 1.5	<0.001

* AVR: Aortic valve replacement; BSA: Body surface area; NYHA: New York Heart Association functional class.

**Table 2:** Expression of circulating microRNAs in the three study subgroups.

MicroRNA Values	Whole Cohort (*n* = 145)	BAV-AR (*n* = 63)	BAV-AS (*n* = 32)	TAV-AS (*n* = 50)	*p*-Value
miR-17	21.8 ± 5.7	17.1 ± 0.8	21.2 ± 1.8	29.0 ± 3.9	<0.001
miR-18a	25.0 ± 4.9	21.8 ± 4.1	24.1 ± 1.5	29.8 ± 3.2	<0.001
miR-19a	23.6 ± 5.3	21.5 ± 1.0	22.5 ± 2.2	26.9 ± 7.7	<0.001
miR-20a	25.5 ± 6.0	20.3 ± 0.8	26.1 ± 4.0	32.0 ± 4.3	<0.001
miR-21	24.1 ± 4.6	20.5 ± 1.0	24.6 ± 3.2	28.3 ± 4.3	<0.001
miR-106a	25.8 ± 5.7	21.0 ± 0.7	28.5 ± 3.7	32.4 ± 3.4	<0.001
miR-145	23.8 ± 3.8	20.7 ± 0.6	26.9 ± 2.5	25.9 ± 4.0	<0.001

CT values ± standard deviation for circulating microRNAs in the three subgroups, *p*-values were calculated for comparison of means using one-way ANOVA between all three subgroups.

**Table 3:** Correlation between maximum aortic diameter and microRNA CT values in the whole study cohort (*n* = 145).

Correlation Coefficient	miR-17	miR-18a	miR-19a	miR-20a	miR-21	miR-106a	miR-145
Aortic diameter (mm)	−0.285	0.044	0.105	−0.215	0.215	−0.221	0.234
*p*-Value	0.005	0.666	0.301	0.031	0.58	0.5	0.1

**Table 4:** Multivariate linear regression model for CT values of miR-17 (*n* = 145).

Variables	Regression Coefficient B	Standard Error	*p*-Value
Aortic valve phenotype (BAV vs. TAV)	−8.521	0.644	0.000
Aortopathy (proximal aorta ≥ 40 mm)	−1.677	0.702	0.019
Gender	0.017	0.554	0.976
Age	0.064	0.018	0.001
Maximal aortic diameter (mm)	0.001	0.029	0.961
